# A Novel Low-Temperature Extrusion Method for the Fused Filament Fabrication of Fluoroelastomer Compounds

**DOI:** 10.3390/mi15050582

**Published:** 2024-04-27

**Authors:** Mookkan Periyasamy, Ronald Campbell, Joey M. Mead, David O. Kazmer, ShibShankar Banerjee, AA Mubasshir, Leeda A. Phaen, Stiven Kodra

**Affiliations:** 1 Greene Tweed & Company LLC., 1684 South Broad St, P.O. Box 1307, Lansdale, PA 19946, USA; rcampbell@gtweed.com; 2 Department of Plastics Engineering, University of Massachusetts, Lowell, MA 01854, USA; joey_mead@uml.edu (J.M.M.); david_kazmer@uml.edu (D.O.K.); shibshankar_banerjee@uml.edu (S.B.); aa_mubasshir@uml.edu (A.M.); leeda_phaen@uml.edu (L.A.P.); 3 Department of Mechanical Engineering, University of Massachusetts, Lowell, MA 01854, USA; stiven_kodra@uml.edu

**Keywords:** additive manufacturing, fused deposition modeling (FDM), curable thermoset elastomer compounds, fluoroelastomer (FKM), perfluoroelastomer (FFKM), buckling force, support tube, filament drive mechanism, precooling, modeling, printing parameters, thermoset elastomer seals

## Abstract

In this work, an additive manufacturing process for extruding fully compounded thermosetting elastomers based on fluorine-containing polymer compositions is reported. Additive manufacturing printers are designed with a dry ice container to precool filaments made from curable fluoroelastomer (FKM) and perfluoroelastomer (FFKM) compounds. A support tube guides the stiffened filament towards the printer nozzle. This support tube extends near the inlet to a printer nozzle. This approach allows low-modulus, uncured rubber filaments to be printed without buckling, a phenomenon common when 3D printing low-modulus elastomers via the fused deposition modeling (FDM) process. Modeling studies using thermal analyses data from a Dynamic Mechanical Analyzer (DMA) and a Differential Scanning Calorimeter (DSC) are used to calculate the Young’s modulus and buckling force, which helps us to select the appropriate applied pressure and the nozzle size for printing. Using this additive manufacturing (AM) method, the successful printing of FKM and FFKM compounds is demonstrated. This process can be used for the future manufacturing of seals or other parts from fluorine-containing polymers.

## 1. Introduction

Additive manufacturing (AM), also commonly referred to as three-dimensional (“3D”) printing, is increasing in popularity in applications such as rapid prototyping and the commercial production of parts. A diverse range of AM processes exist, including vat photopolymerization methods, such as stereolithography (“SLA”); material or binder jetting methods; powder bed fusion methods, such as selective laser sintering (“SLS”); and material extrusion methods, such as fused deposition modeling (“FDM”), fused filament fabrication (“FFF”), and direct pellet extrusion, among others [1,2,3,4,5,6,7,8,9,10].

In material extrusion methods for printing parts such as FDM [7,8], a computer model of a part is generated in which the part is represented as a series of layers. The part is produced by feeding a filament of material to an extruding head, which heats the filament and deposits the heated filament on a substrate to form a layer of the part. Once a layer is formed, the extruding head proceeds to deposit the next layer of the article based upon the computer model of the part. This process is repeated in a layer-by-layer manner until the printed part is fully formed. Similarly, during direct pellet extrusion [9,10], pellets rather than filaments are used as the feed material, and the pellets are fed to an extruding head and are heated and deposited onto the substrate.

A variety of polymeric materials are known for their use in additive manufacturing methods. Common polymeric materials used in additive manufacturing include acrylonitrile butadiene styrene (ABS), polyurethane, polyamide, polystyrene, and polylactic acid (PLA). More recently, high-performance engineering thermoplastics have been used to produce printed articles with improved mechanical and chemical properties relative to common polymer materials. Such high-performance thermoplastics include polyaryl ether ketones, polyphenyl sulfones, polycarbonates, and polyetherimides.

In addition to using the materials as noted above, there have been further attempts to develop techniques using FDM for printing soft thermoplastic elastomers, such as ethylene vinyl acetate (EVA), ethylene–propylene diene monomer in a polypropylene matrix (EPDM + PP), and styrene–ethylene–butadiene–styrene (SEBS) [11,12,13]. However, processing such materials presents challenges when using FDM to form printed parts.

When materials are not chemically crosslinked, they tend to lack an adequate compression set and heat resistance for many applications. To provide better performance, the use of a compounded elastomer (i.e., a curable elastomer composition for vulcanization including a curable polymer, one or more fillers, and a cure system) would be preferable [14,15,16]. As such materials are processed, they form a network structure in the crosslinked rubber system that can negatively impact the ability to fabricate objects using layered FDM technology. Because of their greater heat-resistance and improved compression set, methods to additively manufacture these fully compounded elastomers would enable the production of high-end products, which should exhibit strong interfacial bonding, provided these network structures can be formed successfully with FDM or another additive technology.

Fully compounded curable elastomers pose distinct processing challenges for FFF/FDM 3D printing compared to thermoplastic elastomers (TPEs). These thermosets must remain below their curing temperature to prevent premature crosslinking. However, their inherent high viscosity hinders processing at lower temperatures. Moreover, feeding soft elastomeric filaments through traditional FFF/FDM printers leads to buckling due to their low mechanical rigidity. These challenges converge to make 3D printing fully compounded thermoset elastomers via FFF/FDM a complex undertaking.

Some developments have occurred recently in the additive manufacturing of curable thermoset elastomers, such as Nitrile Butyl Rubber (NBR) [17], Hydrogenated Butyl Rubber (HNBR) [18], and silicones [19,20,21,22,23,24]. However, most reports in the literature employ either the material extrusion approach with custom print heads (a ram-type material extruder, single and twin screw extruder) or the costly vat photopolymerization approach [17,18,19,20,21,22,23,24]. To enable the general consumer to print with high-performance thermoset elastomers, such as NBR, HNBR, silicones, fluoroelastomer (FKM), and perfluoroelastomer (FFKM), it is desirable to find a method that would enable the printing of curable compounds using commercially available 3D printers. Moreover, the ability to print thermoset elastomers in the curable compounded form using commercially available 3D printers will substantially reduce the cost of forming a part using the 3D-printing technique. 

Thermoset elastomers find use in demanding industries like the semiconductor fabrication, downhole tooling, medical devices, aerospace, and automotive industries. These sectors often need specialized, complex parts in small volumes. Traditional compression and injection molding become expensive in these scenarios due to tooling costs and lead times [25]. Additive manufacturing offers a potentially cost-effective alternative.

Seals, gaskets, and other parts produced using FKM and FFKM elastomers are usually recognized for their excellent sealing performance against a wide range of temperatures, pressures, chemicals, and operating environments [26,27,28,29]. Generally, FKM- and FFKM-based seals are expensive and become more expensive when manufactured in low volume. Three-dimensional-printing technology has the potential to be a cost-effective manufacturing process for low-volume FKM- and FFKM-based seals or components. However, there are very few reports on the 3D printing of FKM and FFKM compounds in the literature [30]. This paper reports the preliminary outcomes of this group’s efforts to overcome the challenges of 3D printing parts from FKM and FFKM filaments.

## 2. Experimental Section: Materials and Methods

### 2.1. Precursors and Material Preparation

Two curable fluoropolymer compounds were evaluated for additive manufacturing, also known as 3D Printing. Formulations of these two compounds are reported in Table 1. The first compound was a peroxide-cured fluoroelastomer (FKM) compound. The rubber stock of an FKM compound, Tecnoflon^®^ 959, was purchased from Solvay (West Deptford, NJ, USA). A peroxide system was selected from the group consisting of 2,5-dimethyl-2,5-di(t-butylperoxy) hexyne-3, 2,5-dimethyl-2,5-di(t-butylperoxy) hexane, dicumyl peroxide, dibenzoyl peroxide, t-butyl perbenzoate, α,α′-bis(t-butylperoxy-diisopropylbenzene), and di[1,3-dimethyl-3-(t-butylperoxy)-butyl] carbonate [31]. Peroxide was procured from R.T. Vanderbilt Inc. (Norwalk, CT, USA). The second compound was a bis-phenyl-cured perfluoroelastomer (FFKM). FFKM rubber stock was obtained from Greene Tweed (Lansdale, PA, USA). 2,2-bis(3 amino-4-hydroxyphenyl) hexafluoro propane, also known as BOAP, was used as a curative, and it was purchased from TCI America (Portland, OR, USA). Fumed silica was used as a filler in both compounds and was purchased from Fiberglass Supply Inc. (Burlington, WA, USA). Mineral fillers such as SiO_2_ and aluminum silicate exhibit high plasma resistance, and they can shield the FKM and FFKM polymers in the seals [32]. The compounding or mixing of formulation ingredients was performed in a Brabender Plasticorder Intelli-Torque Plus (Model 01-55-000, Duisburg, Germany) internal mixer with counter-rotating screws. A Brabender single-screw extruder was used to prepare filaments from FKM and FFKM compounds which had two different nominal outer diameter (OD) sizes, namely 1.75 mm and 2.7 mm, respectively.

### 2.2. Three-Dimensional Printing

**Figure 1:**
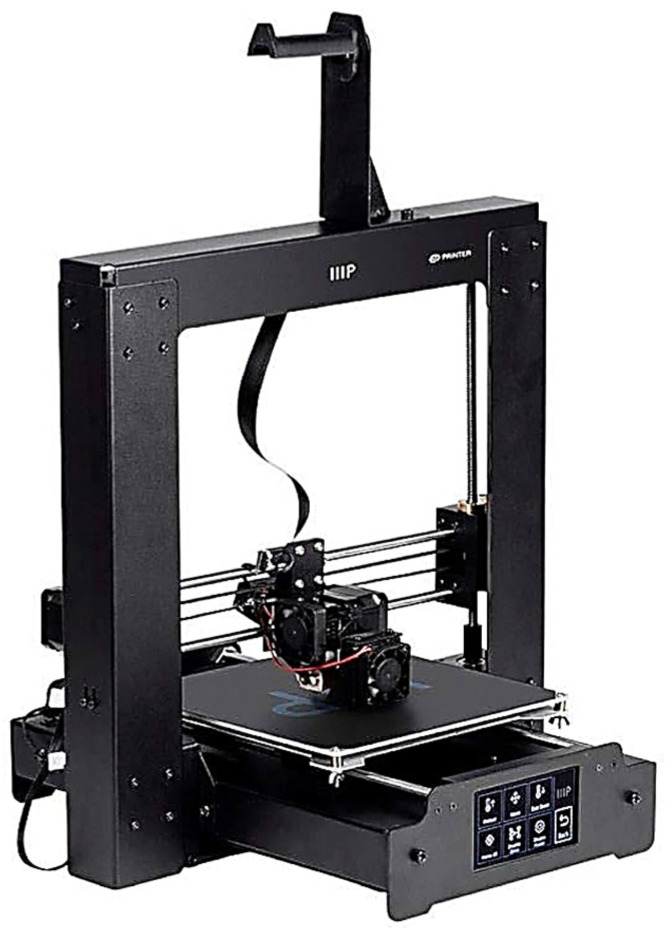
A Monoprice Maker Select Plus FDM printer which was modified and used for FKM and FFKM 3D printing.

A low-cost FDM 3D printer, Maker Select Plus 3D (Figure 1), manufactured by Monoprice (USA), was used for 3D printing. The stock print head of the Maker Select Plus printer was swapped with a HEMERA E3D XS extruder [33,34] to lower the free column length below the filament drive wheels and the hot end. A custom precooling unit was further integrated with the HEMERA extruder. This unit consisted of a metal enclosure thermally insulated on five sides to minimize heat transfer to the ambient environment. Prior to printing, the metal enclosure was filled with dry ice. The reasoning behind and outcome of this modification are discussed in detail in Section 3.

The print head was set to a layer height of 1 mm, a line width of 1.75 mm, an infill density of 100%, a build plate temperature of 25 °C, and a print speed of 4 mm/s. Samples were run using a nozzle orifice diameter of 1.5 mm and a nozzle temperature of 80 °C. The print temperature was maximized to lower the viscosity sufficiently, allowing easier extrusion from the nozzle and encouraging adhesion to a frictional surface formed using an adhesive tape material positioned on the substrate surface. The printing temperature of FKM and FFKM compounds was kept below their curing temperature, which were determined based on Dynamic Mechanical Analyzer (DMA) and Differential Scanning Calorimeter (DSC) analyses.

Tensile bars were successfully printed from the materials according to the guidelines set by the American Society for Testing and Materials (ASTM) D412-C [35].

### 2.3. Thermal and Mechanical Analysis

Thermal analyses of FKM and FFKM compounds were carried out using DSC (TA-Instruments DSC 250, New Castle, DE, USA) to determine the suitable temperature range for printing with the FKM and FFKM filaments. Uncured rubber samples were heated from 25 °C to 200 °C at a 10 °C/min rate, followed by cooling to 25 °C at 5 °C/min and then heated again to 200 °C at a rate of 10 °C/min. Dynamic mechanical analysis was conducted on uncured samples using DMA (TA Instruments DMA Discovery 850, New Castle, DE, USA), and tests were conducted in tension mode. DMA analysis was run at low temperatures from −80 °C to 20 °C at a heating rate of 3 °C/min and at a 50 g force/load. Cooling was achieved using liquid nitrogen. A compound sample was prepared as an O-ring with a 139 in./diameter ratio using an uncured material.

## 3. Results and Discussion

### 3.1. Addressing Challenges in FKM and FFKM Rubber Additive Manufacturing

During the initial 3D printing experimental trials using the low-cost Monoprice Maker Select Plus FDM printer and the nominal 1.8 mm or 2.8 mm OD filaments made from FKM and FFKM compounds, four major issues were identified. They were (1) filament clogging in the nozzle, (2) filament buckling, (3) under-extrusion, and (4) poor bed adhesion. Each of the four issues was subsequently addressed.

#### 3.1.1. Issue 1: Nozzle Clogging

**Figure 2:**
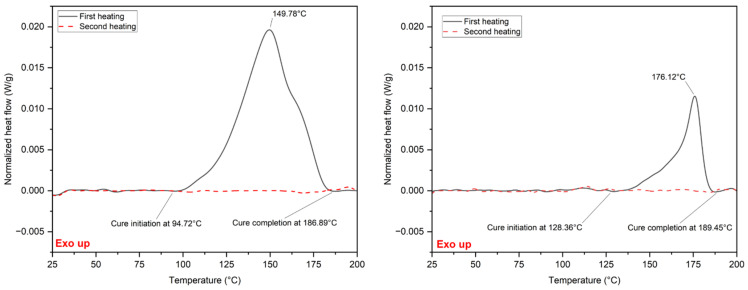
DSC trace of FKM (**left**) and FFKM (**right**) compounds.

Printing parts from FKM or FFKM filament requires feeding the filament and then heating the filament until it is soft enough to extrude through the nozzle. The root cause of filament clogging the nozzle was the premature curing of the FKM or FFKM filament during the printing of parts. The issue was solved by heating the filament to a temperature that was sufficient to increase the flow of the curable fluoropolymer composition within the printer apparatus without exceeding its curing temperature. To identify the curing initiation temperature for the FKM and FFKM compounds from which the filament is made, thermal analysis of the compounds was carried out using a DSC. DSC analysis identified a curing initiation temperature of ~95 °C and a curing completion temperature of ~187 °C for the FKM compound. DSC analysis also identified the curing initiation and completion temperatures of the FFKM compound, which were ~128 °C and ~189 °C, respectively. The DSC results are shown in Figure 2. Three-dimensional printing of FKM and FFKM compound filaments was carried out at 130 °C and 150 °C, respectively, which was between the filaments’ onset of curing, but below their curing temperature. This approach solved the nozzle-clogging issue.

#### 3.1.2. Issue 2: Filament Buckling

**Figure 3:**
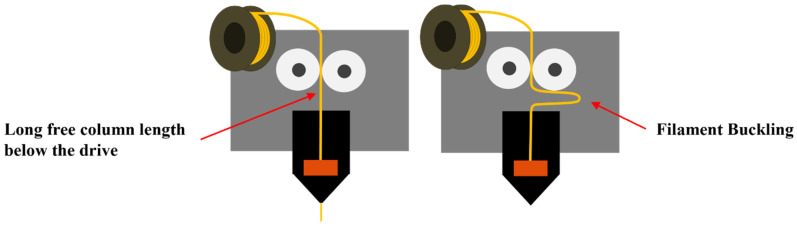
Buckling of FKM and FFKM compound filament.

When the FKM or FFKM filament with a 1.8 mm or 2.8 mm outside diameter (OD) was driven through the hot end of the printer by the motor drive mechanism to print the seal or other parts, the filament buckled, as demonstrated by illustrations in Figure 3, which resulted in a failure to print the seal or part. The primary root causes of filament buckling were (a) the printer design having too long a “free column length” below the filament drive wheels and the hot end and (b) the filaments not having sufficient mechanical strength/rigidity because of their elastic nature.

**Figure 4:**
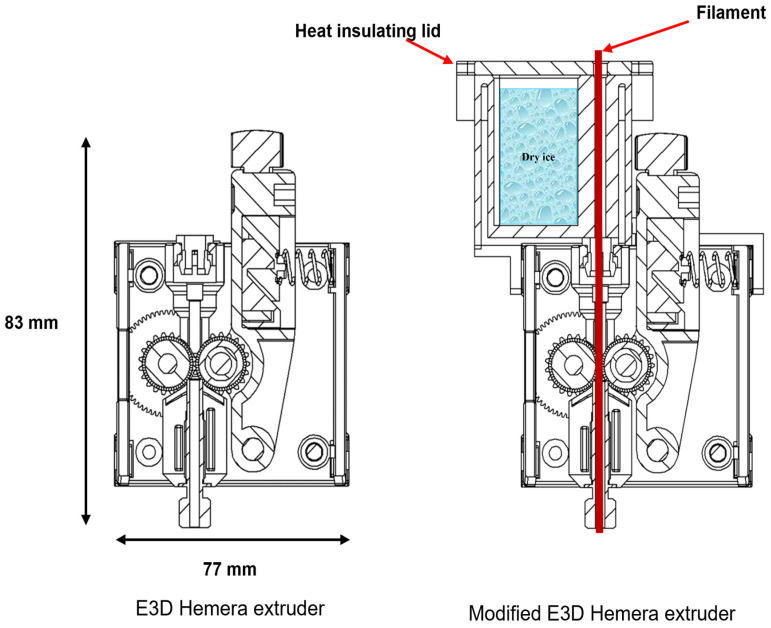
E3D Hemera extruder: (**left**) as is [33,34] and (**right**) modified with dry ice precooler.

To reduce the free column length, the print head of the Monoprice Maker Select Plus was replaced with a HEMERA E3D extruder commercialized by E3D [33,34]. This print head, or extruder, is very compact, relatively inexpensive (~USD 200), and can be mounted to any 3D printer. Most importantly, the Hemera extruder has one of the shortest and most constrained filament paths produced for a 3D printer extruder. The “free column length”, or the distance between the filament drive wheels and the hot end, was about 5 mm for the Hemera extruder. The 3:1 gear ratio dual drive gear also provided high torque, very good grip, and precise stepping control of the driven filament. Cross-sections of the Hemera extruder before [33,34] and after modification are shown in Figure 4.

#### 3.1.3. Issue 3: Under-Extrusion

After minimizing the “free column length”, under-extrusion of the FKM and FFKM filaments was observed when printing seals and other parts. We hypothesized that the under-extrusion was caused due to using a 0.2 mm nozzle for a very high-viscosity material, so this was replaced with a much larger-diameter nozzle (2 mm orifice). We found that even using a 2 mm nozzle did not solve either the under-extrusion or buckling issues. Further root cause analyses were carried out to identify the reason for the under-extrusion. It was identified that the primary reason for the under-extrusion was that the print head’s drive gears failed to apply sufficient pressure to the filament. This lack of applied pressure was attributed to the elastic and soft nature of the FKM and FFKM filaments. DMA runs revealed that precooling the filaments using dry ice and a custom configuration, as shown on the right in Figure 4, could stiffen and increase the rigidity of the FKM or FFKM filaments between the drive gears and heated nozzle, which in turn could significantly increase the maximum pressure that could be applied to the filaments without causing buckling.

**Figure 5:**
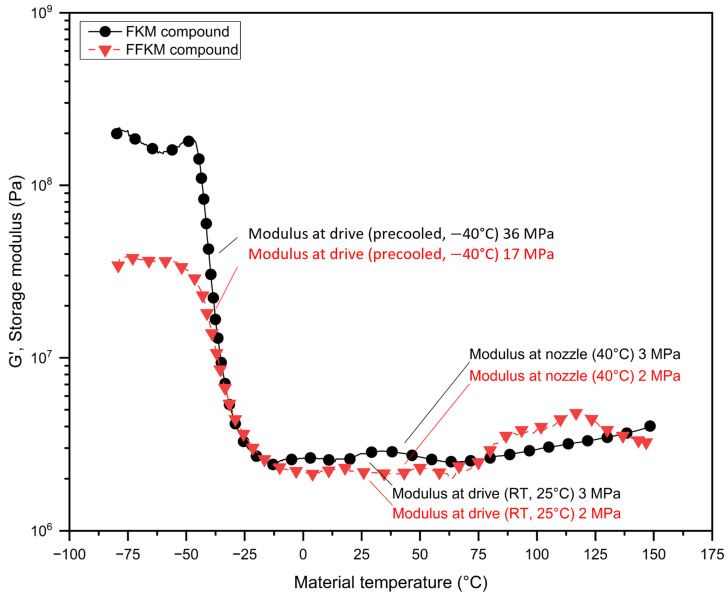
DMA trace for FKM and FFKM filament to determine storage modulus; here, RT refers to room temperature.

An infrared temperature gun revealed the filament temperature was about −40 °C when it reached the drive gear wheels after passing through the passage adjacent to the dry ice chamber. The temperature sweep from −80 °C to 150 °C using a DMA machine provided the storage modulus of the FKM and FFKM filaments as a function of temperature. The results are reported in Figure 5. Without the precooling configuration, the filaments entered the drive wheels at room temperature and the storage moduli of the FKM and FFKM filaments at room temperature were 3 and 2 MPa, respectively. On the other hand, at −40 °C, the temperature at which the filaments entered the drive wheels using the precooling configuration, the storage moduli of the FKM and FFKM filaments were 36 and 17 MPa, respectively. The higher modulus of the FKM filament can be attributed to the higher loading of the reinforcing filler in the FKM compound. Using the Euler equation to determine the critical buckling load of a long column (shown in the fifth row, last column of Table 2) [36], the critical buckling loads for the filaments were calculated and reported in Table 2. Here, F is the critical buckling load, $E_{d}$ is the storage modulus of filaments at the drive wheel temperature, I is the moment of inertia of the circular filament, μ is the constraint factor (μ=1 here), and L is the effective length of the column (5 mm in this case). Based on the critical buckling load, the maximum pressure that can be applied to the filaments without causing buckling was calculated and reported in Table 2. As shown in Table 2, precooling allowed us to apply fourteen and seven times more pressure to the FKM and FFKM filaments without causing buckling compared to the usual configuration.

Furthermore, the maximum draw ratio and minimum nozzle orifice diameter for the FKM and FFKM filaments were calculated using expressions given by Hoffman and Sachs for frictionless wire drawing processes through a die (shown in the seventh row, in the last column in Table 2) [37,38]. Here, F is the applied load, $A_{0}$ is the diameter of the filament, $A_{f}$ is the diameter of the nozzle outlet, and $E_{n}$ is the storage modulus at the nozzle temperature. An infrared temperature measurement indicated that the nozzle temperature was about 40 °C. Hence, the storage modulus at 40 °C was used to calculate the maximum draw ratio and minimum nozzle orifice diameter for the FKM and FFKM filaments, which is reported in Table 2.

As shown in Table 2, dry ice cooling increased the storage modulus by more than eight-fold for both the FKM and FFKM compounds. This approach of cooling the rubber filaments before printing and thus increasing the rigidity allowed enough pressure (14× for FKM and 7× for FFKM compared to the usual configuration) to be translated through the filaments and thus solved the under-extrusion issue.

#### 3.1.4. Issue 4: Poor Bed Adhesion

Cooling filaments to subzero temperatures increased the filament rigidity and allowed a stable flow of the material through the extruder nozzle. Upon further trials of 3D printing, poor bed adhesion of the extruded material became apparent. Multiple approaches were tested, such as using rough surfaces, adhesives, and a heated print bed to solve the issue. Double-sided adhesive tape showed the best result. The first layer of print adhered properly with the adhesive tape and allowed for continuous printing.

### 3.2. Identifying Optimal 3D-Printing Parameters for FKM and FFKM Rubber Filaments

**Figure 6:**
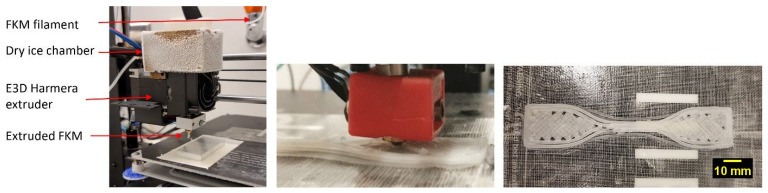
**Left**—printer setup used for FKM and FFKM printing; **middle**—printing of an ASTM D412 type C tensile bar; **right**—printed ASTM D412 type C tensile bar from an FFKM compound.

Finally, using the approaches discussed earlier to tackle the four key challenges of FKM and FFKM rubber printing, we succeeded in 3D printing parts from the FKM and FFKM filaments. The printing parameters are reported in Table 3. The printer setup, printing process, and a printed tensile bar are shown in Figure 6.

## 4. Conclusions

The 3D printing of fully compounded elastomers is challenging for a variety of reasons, including the low modulus of the material, which can lead to buckling in the printer, and the need to ensure the compounded elastomer remains below the curing temperature during printing. Multiple issues were encountered during 3D printing experiments with FKM and FFKM elastomeric filaments. Nozzle clogging was addressed by lowering the nozzle temperature to avoid any curing during the printing process. Filament buckling and under-extrusion due to the low modulus were addressed by a precooling setup that raised the modulus sufficiently to avoid buckling and by reducing the free column length. Ultimately, the printing process for filaments made from thermoset FKM and FFKM was performed with a modified FDM 3D printer by mounting a Hemara extruder and using a dry ice precooler. DMA and DSC thermal analyses were used to select the temperatures for printing. Modeling studies helped us to calculate the maximum applied pressure and buckling force that could be used to print without buckling of the filament. A very small number of preliminary experimental samples were printed for demonstration; the scope remains on examining the characteristics of the cured FKM and FFKM printed specimens after thermal cure. This process could be used to make printed parts such as O-Rings, gaskets, and other complicated components. The information reported in this paper is also included in a pending patent by Greene Tweed & Company [30].

## Figures and Tables

**Table 1:** Formulation of FKM and FFKM rubber.

Element	Component	Weight Percentage (%)	
		FKM Compound	FFKM Compound
**Elastomer**	FKM	74.08	-
	FFKM	-	98.72
**Curative**	Peroxide cure package	3.70	-
	BOAP	-	1.28
**Filler**	Silica	22.22	-

**Table 2:** Estimated modulus and calculated buckling force.

Properties	FKM Compound		FFKM Compound		Source
	RT	Precooled	RT	Precooled	
**A. Material properties**					
Modulus at drive, $E_{d}$ (MPa)	3	36	2	17	DMA results from Figure 5
Modulus at nozzle, $E_{n}$ (MPa)	3	3	2	2	
**B. Filament geometry**					
Filament diameter, $A_{0}$ (mm)	1.75	1.75	1.75	1.75	N/A
Free column length, *L* (mm)	5	5	5	5	N/A
**C. Analysis**					
Buckling force, *F* (N)	0.5	6.5	0.4	3.1	$F=\frac{\mu E_{d}I\pi^{2}}{L^{2}}$
Buckling pressure, *P* (MPa)	0.2	2.7	0.2	1.3	$P=\frac{F}{\pi A_{0}^{2}/4}$
Maximum draw ratio, $A_{0}/A_{f}$ (mm/mm)	1.1	2.5	1.1	1.9	$F=A_{0}\;E_{n}\ln\left(\frac{A_{0}}{A_{f}}\right)$
Minimum nozzle diameter, $A_{f}$ (mm)	1.7	1.1	1.7	1.3	$F=A_{0}\;E_{n}\ln\left(\frac{A_{0}}{A_{f}}\right)$

RT—room temperature.

**Table 3:** Printing parameters used while FKM and FFKM 3D printing.

Print Parameters	Values
Nozzle size	1.6 mm (tapered)
Print temperature	150 °C
Cooler temperature	−60 °C
Nozzle temperature	40 °C
Print speed	7 mm/s
Layer height	1 mm
Line width	1.75 mm
Infill density	100%
Adhesive on bed	yes

## Data Availability

Data are contained within the article.
